# Novel Bioplastic Based on PVA Functionalized with Anthocyanins: Synthesis, Biochemical Properties and Food Applications

**DOI:** 10.3390/ijms25189929

**Published:** 2024-09-14

**Authors:** Giuseppe Tancredi Patanè, Antonella Calderaro, Stefano Putaggio, Giovanna Ginestra, Giuseppina Mandalari, Santa Cirmi, Davide Barreca, Annamaria Russo, Teresa Gervasi, Giovanni Neri, Meryam Chelly, Annamaria Visco, Cristina Scolaro, Francesca Mancuso, Silvana Ficarra, Ester Tellone, Giuseppina Laganà

**Affiliations:** 1 Department of Chemical, Biological, Pharmaceutical and Environmental Science, University of Messina, 98166 Messina, Italy; giuseppe.patane@studenti.unime.it (G.T.P.); anto.calderaro@gmail.com (A.C.); stefano.putaggio@studenti.unime.it (S.P.); giovanna.ginestra@unime.it (G.G.); giuseppina.mandalari@unime.it (G.M.); santa.cirmi@unime.it (S.C.); francesca.mancuso@unime.it (F.M.); ester.tellone@unime.it (E.T.); giuseppina.lagana@unime.it (G.L.); 2 Department of Biomedical and Dental Sciences and Morphofunctional Imaging, University of Messina, 98125 Messina, Italy; teresa.gervasi@unime.it; 3 Engineering Department, University of Messina, 98166 Messina, Italy; giovanni.neri@unime.it (G.N.); chelly.meryam@gmail.com (M.C.); annamaria.visco@unime.it (A.V.); cscolaro@unime.it (C.S.); 4 Laboratory of Toxicology-Microbiology Environmental and Health, LR17ES06, Sfax 3038, Tunisia; 5 Institute for Polymers, Composites and Biomaterials, CNR-IPCB, Via Paolo Gaifami 18, 95126 Catania, Italy

**Keywords:** food packaging, active packaging, circular economy, polyphenols, anthocyanins, biodegradable, polyvinyl alcohol

## Abstract

Over the last ten years, researchers’ efforts have aimed to replace the classic linear economy model with the circular economy model, favoring green chemical and industrial processes. From this point of view, biologically active molecules, coming from plants, flowers and biomass, are gaining considerable value. In this study, firstly we focus on the development of a green protocol to obtain the purification of anthocyanins from the flower of *Callistemon citrinus*, based on simulation and on response surface optimization methodology. After that, we utilize them to manufacture and add new properties to bioplastics belonging to class 3, based on modified polyvinyl alcohol (PVA) with increasing amounts from 0.10 to 1.00%. The new polymers are analyzed to monitor morphological changes, optical properties, mechanical properties and antioxidant and antimicrobial activities. Fourier transform infrared spectroscopy (FTIR) spectra of the new materials show the characteristic bands of the PVA alone and a modification of the band at around 1138 cm^−1^ and 1083 cm^−1^, showing an influence of the anthocyanins’ addition on the sequence with crystalline and amorphous structures of the starting materials, as also shown by the results of the mechanical tests. These last showed an increase in thickening (from 29.92 μm to approx. 37 μm) and hydrophobicity with the concomitant increase in the added anthocyanins (change in wettability with water from 14° to 31°), decreasing the poor water/moisture resistance of PVA that decreases its strength and limits its application in food packaging, which makes the new materials ideal candidates for biodegradable packaging to extend the shelf-life of food. The functionalization also determines an increase in the opacity, from 2.46 to 3.42 T%/mm, the acquisition of antioxidant activity against 2,2-diphenyl-1-picrylhdrazyl and 2,2′-azino-bis-(3-ethylbenzothiazoline-6-sulfonic acid) radicals and, in the ferric reducing power assay, the antimicrobial (bactericidal) activity against different *Staphylococcus aureus* strains at the maximum tested concentration (1.00% of anthocyanins). On the whole, functionalization with anthocyanins results in the acquisition of new properties, making it suitable for food packaging purposes, as highlighted by a food fresh-keeping test.

## 1. Introduction

In the European Union, it is estimated that more than 2.5 billion tons of by-products are produced each year [1]. Consequently, legislation on the management of these by-products is directed towards promoting the transition to a circular economy, as an alternative to the linear economic model. The circular economy is a production and consumption model that involves sharing, lending, reusing, repairing, reconditioning and recycling existing materials and products for as long as possible [2,3]. Over the past two decades, attempts have been made to apply this model to all sectors [4]. In particular, the production of plastics is becoming an increasingly important issue worldwide due to their widespread applicability. In fact, they are used in packaging, food packaging, the construction industry and the automotive field, requiring the production of more than 400 million tons of plastics per year [5,6]. This enormous production results in approximately 353 million tons of plastic waste, about 40% of which comes from packaging plastics [7]. The main problem is that today, about 90% of these wastes are fossil-based and non-biodegradable plastics, which are incinerated, causing environmental pollution, and often end up in the oceans, generating microplastics harmful to marine animals [8]. In this perspective, to support a circular economy plastic model, the use of biodegradable materials to produce bioplastics seems to be the only approach to reduce environmental pollution and the problems caused by fossil-based plastics [9,10]. Nowadays, with adherence to green chemistry and circular economy principles, the most-used materials for the production of bioplastics include natural compounds derived from the extraction of biomass (e.g., cellulose, starch and gelatin), polymers derived from alcoholic fermentation processes that are not biodegradable (e.g., polyethylene), synthetic polymers that are degradable (e.g., polyvinyl alcohol) or biopolymers derived from bacterial processes (e.g., polylactic acid and polyhydroxyalkanoates) [11,12,13]. 

Today, researchers’ efforts aim to combine the desire to produce active food packaging or functionalized nanomaterials with the necessity of using biodegradable materials or materials from renewable sources. In fact, the opportunity to incorporate biologically active compounds within bioplastics makes it possible to obtain active packaging, which is able to release the incorporated compounds, so as to have new anti-oxidant and anti-microbial properties, or to increase food preservation by counteracting the formation of free radicals and bacterial growth without changing the initial mechanical and physical properties too much [14,15,16]. Moreover, the possibility of utilizing waste products to develop an efficient commercial-scale process is one of the main results to obtain following direct utilization or after bacterial fermentation processes. These waste products are often a rich source of biological active compounds, such as phenols and polyphenols, able to act as antimicrobial agents and, at the same time, having health-promoting properties, based on the basic characteristics of their skeletons and the different patterns of substitutions present. 

In this frame, a polymer that is a good candidate for active food packaging is PVA. It is a synthetic polymer, obtained by the partial or complete hydrolysis of polyvinyl acetate, biodegradable and water-soluble with good thermal and mechanical properties [17,18,19]. These excellent qualities make PVA a material widely used today in various industries, such as medical, pharmaceutical, textile and food. It was reported that in 2016, around 31.4% of the PVA produced was directed towards the production of food packaging [20]. On the other hand, over the last decade, there has been a growing interest by the scientific community in natural compounds and extracts rich in polyphenols, commonly considered waste products from the food and flower industries [21,22]. Among these, the use of anthocyanins as molecules rich in numerous biological properties and as natural colorants has recently caught the attention of researchers [23,24]. Our research group and others have reported that *Callistemon citrinus* sp., an ornamental plant found in the Mediterranean area, is characterized by a phytocomplex that is particularly rich in polyphenols and, in particular, anthocyanins, which are endowed with numerous biological activities [25]. On this basis, the aim of this work was to produce PVA-based bioplastics functionalized with four anthocyanins purified from *Callistemon citrinus* flowers (a waste product of floriculture industries), applying a green approach based on prediction and response surface methodology, and analyze them in terms of structural, optical, mechanical, antioxidant and antimicrobial properties to assess their potential application as food packaging, tested also by a food fresh-keeping test.

## 2. Results and Discussions

### 2.1. Response Surface Optimization and Verification of Predictive Model

**Figure 1:**
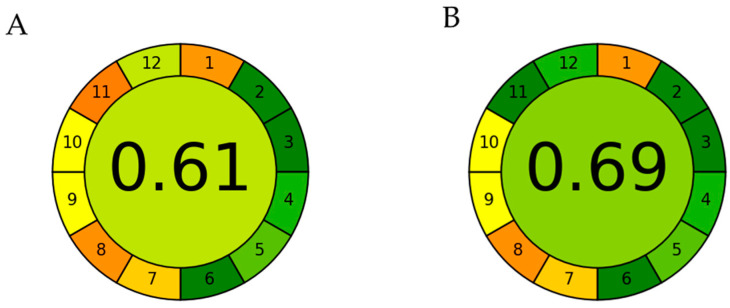
Representative pictogram for the green index of the extraction of anthocyanin from *Callistemon citrinus* using two different methods. (**A**) The index for the extraction with accelerated solvent extraction using methanol solution; (**B**) The index for the extraction with MAE using the new ethanol (EtOH) solution. In both pictograms, the colour scale (red-yellow-green) indicates the performance at each stage of the procedure. The less chemically ‘green’ the process, the more red it appears.

One of the goals of the United Nations 2030 Agenda is to increase and promote green extraction techniques, utilizing unconventional extraction techniques and green solvents, resulting in less hazardous processes and reducing environmental impacts. As part of these efforts, several approaches have been developed to increase and ameliorate the environmentally friendly extraction processes to efficiently convert diverse biomass wastes into valuable and economically important products such as bioactive compounds and biobased materials. In this regard, the program AGREE (Analytical GREEnness Metric Approach and Software), will be employed to analyze the green index of the extraction and optimize each step to decrease the impact by energetic requirement reduction, waste production, miniaturization, automation and solvent utilization [26]. The Analytical GREEnness calculator utilizes a flexible, comprehensive and straightforward assessment approach to supply an easily interpretable and informative result as a pictogram indicating the final score (Figure 1). The assessment criteria are taken from the 12 principles of green extraction methodology and are transformed into a unified 0–1 scale. After the selection of the steps of the procedures, we will perform the separation and identification of the bioactive compounds by RP-HPLC-DAD or, if possible, by spectroscopic techniques. 

**Figure 2:**
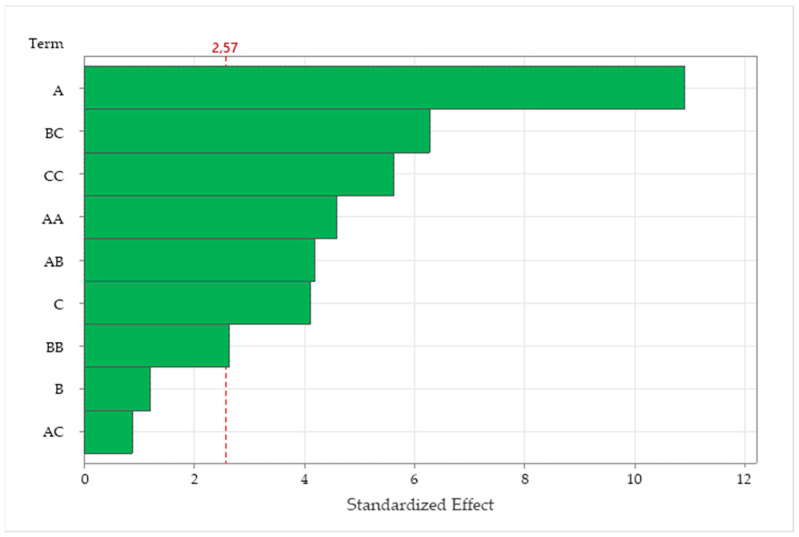
Pareto chart diagram for the extraction of total anthocyanin content (TAC) from *Callistemon citrinus*. A = microwave power (W); B = extraction time (min); C = EtOH% in the extraction solution.

The results of these procedures will be utilized, in a predictive approach, to optimize and maximize the yield with response surface methodology (RSM) modeling and optimization. Response surface methodology is an efficient modeling tool, able to solve linear and non-linear multivariate regression problems and, through simulation, optimize complex processes based on more-efficient and easier arrangements and interpretations of experiments, compared to other traditional methods [27,28,29]. The utility of this approach, which is less laborious and time-consuming than other methods, makes it suitable, in terms of advantages, for the extraction of several natural components, such as phenolics, chromones, saponins and polysaccharides [29,30,31,32,33]. Fifteen experiments were conducted in randomized order, according to a Box–Behnken design, to study the interactions and impacts of three independent variables (microwave power, extraction time and percentage of solvent) on the efficiency of MAE extraction of anthocyanins from *Callistemon citrinus*. Total anthocyanin content (TAC) was considered to evaluate the best MAE conditions for obtaining anthocyanins. Table 1 shows the yields obtained under the different conditions evaluated. Anthocyanin concentrations ranged from 197.625 to 265.280 mg/g. In general, at higher concentrations of ethanol in the extraction solutions, there was a lower concentration of extracted anthocyanins. The response surface methodology was followed to calculate the TAC coefficients in the proposed quadratic model and to estimate the statistical significance of the regression coefficients. As we can see in the Pareto chart (Figure 2), the most influential parameter turns out to be the power of the microwaves, then the combination of minutes and the percentage of ethanol, and, finally, the % of ethanol used within the extraction mixture.

### 2.2. Statistical Analysis and Model Fitting

Multiple regression analysis was performed, and the dependent variables based on the results in Table 1 were related by the following second-order polynomial equations.

$$\begin{array}{l} TAC=224.0+0.3887Mw-13.87Minutes+0.575EtOH-0.000980{Mw}^{2}\\ \;\;\;\;\;\;\;+0.352{Minutes}^{2}-0.01921{EtOH}^{2}\\ \;\;\;\;\;\;\;+0.02149MwxMinutes+0.000719MwxEtOH\\ \;\;\;\;\;\;\;++0.1287MinutesxEtOH \end{array}$$

The results of statistical tests using analysis of variance (ANOVA) are presented in Table 2. The significance of each coefficient was assessed using the *p*-value, which could reveal the interaction behavior between the variables. TAC was significantly dependent on MW, which had a positive linear effect. In addition, from the other individual parameters, EtOH % has a high significance, in contrast to minutes alone. Regarding the cross-product between the various independent variables, the coefficients of MW × M and M × EtOH % have a high significance (*p* < 0.005). In particular, the regression analysis model for TAC shows that the R-sq value was 98.08% and the adjusted and predicted R-sq values were 94.64% and 70.77%, respectively. The high values obtained indicate a good explanatory ability with the fitted model, suggesting that TAC can be considered a useful dependent response for predicting the best extraction conditions in this condition.

### 2.3. Optimization of MAE Conditions 

**Figure 3:**
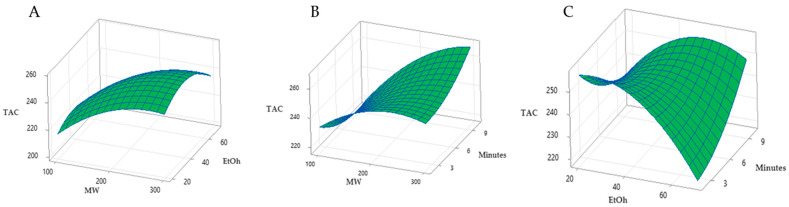
Response surface plots analysis for the total anthocyanin content yield (TAC) from *Callistemon citrinus* powder with microwave assisted extraction (MAE) with the same solid-liquid ratio, 1:10 (*w*/*v*). (**A**) microwave power and EtOH % in the reaction mix; (**B**) microwave power and minutes; (**C**) EtOH % in the reaction mix and minutes.

In order to clearly see the relationship between two independent variables and the TAC response, we used three-dimensional (3D) surface diagrams. The results expressed in the Figure 3 clearly show how the anthocyanin yield, expressed via TAC levels, increases linearly with increasing MW power and decreases with increasing EtOH % in the reaction solution. 

In order to optimize the proposed extraction model, using the values obtained from the polynomial equation, we used the values proposed by the software, 300 MW, 10 min and 53.84% EtOH, as extraction conditions, thus obtaining a theoretical yield of 269.55 mg of anthocyanins. We validated the conditions proposed by the system and obtained a yield of approximately 268.5 ± 4.7 mg/g of cyanidin-3,5-*O*-diglucoside.

### 2.4. RP-HPLC-DAD Identification of the Obtained Anthocyanins

The anthocyanins obtained with the above-described protocol were separated and identified by RP-HPLC-DAD. The chromatographic separation showed the presence of only four compounds, in accordance with the data reported by Laganà et al. [25]. Moreover, the inspection of the chromatograms at different wavelengths (250, 280, 325, 517) showed the absence of other compounds. The four anthocyanins were identified by retention time (Rt), inspection of their UV–visible spectrum, comparison with commercial standards and comparison with the literature. On this basis, the compounds were identified as: cyanidin-3,5-*O*-diglucoside (cyanin, Rt 17.6 min), peonidin-3,5-*O*-diglucoside (peonin, Rt 20.2 min), cyanidin-3-*O*-glucoside (Rt 22.8 min) and cyanidin-coumaroylglucoside-pyruvic acid (Rt 25.0 min). On the whole, cyanidin-3,5-*O*-diglucoside and peonidin-3,5-*O*-diglucoside were, by far, the most abundant compounds, accounting for ~90% of the compounds identified (~56 and ~34%, respectively).

### 2.5. Preparation and Characterization of PVA-Based Bioplastic with Anthocyanins

Starting from the materials obtained from the above-described procedure, we utilized the powder of anthocyanins to produce bioplastics by combining them with PVA to obtain the final concentrations corresponding to 1.0, 0.5, 0.25 and 0.10% of anthocyanins. Each bioplastic appeared macroscopically similar and homogeneous, with a different color depending on the total amount of anthocyanins. The analysis of their functional and biological characteristics is described below.

#### 2.5.1. FTIR

**Figure 4:**
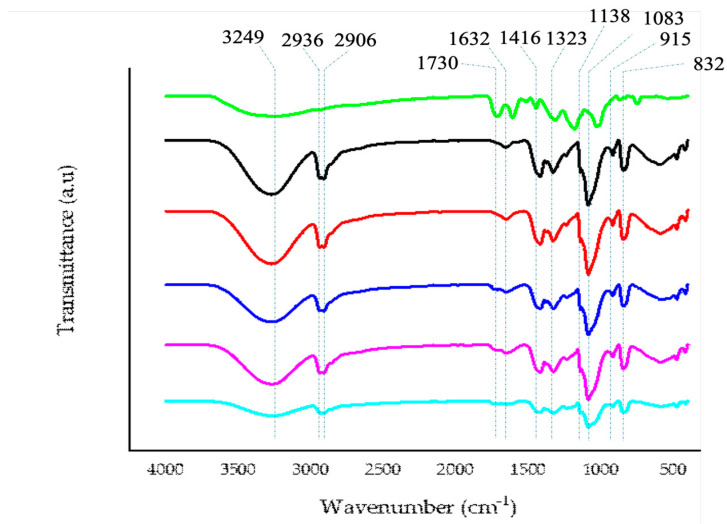
Representative FTIR spectra of the new PVA-based bioplastics produced by the addition of increasing amount of anthocyanins (0.0–1.0%). (**―**) Anthocyanins powder; (**―**) PVA alone; (**―**) PVA plus 0.1% anthocyanins; (**―**) PVA plus 0.25% anthocyanins; (**―**) PVA plus 0.5% anthocyanins; (**―**) PVA plus 1.0% anthocyanins.

In Figure 4 the characteristic FTIR spectrum of PVA, characterized by bands with a maximum at around 3249 cm^−1^ (due to O—H stretching), 2936 cm^−1^ (due to asymmetric stretching of CH_2_), 2906 cm^−1^ (due to symmetric stretching of CH_2_), 1632 cm^−1^ (due to water absorption), 1416 cm^−1^ (due to CH_2_ bending), 1323 cm^−1^ (due to δ (OH), rocking with CH wagging), 1138 cm^−1^ (due to shoulder stretching of C–O, representative of a sequence with a crystalline structure), 1083 cm^−1^ (due to stretching of C=O and bending of -OH, representative of a sequence with an amorphous structure), 915 cm^−1^ (CH_2_ rocking), 832 cm^−1^ (C–C stretching) is depicted. As can be seen from the graph, the modification of the starting materials with increasing concentrations of anthocyanins from 0.1 to 1.0% does not induce the appearance of a new band, indicating a dispersion of utilized materials to functionalize the new bioplastic, and the appearance of a new band at the maximum utilized concentration at about 1730 cm^−1^ is due to the increasing concentration of anthocyanins. There are also some changes in the bands at 1138 cm^−1^ and 1083 cm^−1^, influencing the sequence with crystalline and amorphous structures that are directly related to the increasing percentage of anthocyanins added, in accordance with the obtained mechanical data.

#### 2.5.2. Optical Properties

One of the most important parameters of food packaging plastics is transparency. We evaluated both transparency and opacity, which can determine the amount of UV–visible light that can pass through plastic. Bioplastics functionalized with various % of anthocyanins, become homogeneously more orange-red, compared to PVA alone, due to the presence of anthocyanins. As shown in Table 3, PVA alone has a high transparency of 90%, making it a very transparent material. According to the literature, when the value is between 10 and 80%, one speaks of translucent materials, while values are above 90%, as in the case of PVA alone, one speaks of highly translucent materials [34]. In the samples that are functionalized, there is a decrease in transmittance, which is proportional with the % of anthocyanins dispersed within the sample, until we have a T-value of 78.91% with the highest concentration of anthocyanins. The opacity levels also confirm that in direct proportion to the anthocyanin concentrations found in the sample, the bioplastics become more opaque, becoming more protective against light and radiation that may favor lipid peroxidation processes. It goes from a value of 2.46 (T%/mm) for PVA alone, up to 3.42 for the most functionalized bioplastic.

In the case of the color change, the colorimetric and non-colorimetric results clearly show that the anthocyanins were homogeneously distributed within the PVA during the casting process, as there is a clear color change from PVA to PVA with 1% of anthocyanins. In detail, the data show that L* decreases sharply, in contrast to a* and b*, which increase, as also confirmed by data in the literature. Especially for B, there is a linear and progressive increase, showing how anthocyanins behave as yellow–orange dyes(Table 4).

### 2.6. Mechanical Properties 

When comparing the physical–mechanical properties of PVA-based bioplastics (Table 5) with different concentrations of anthocyanins with pure PVA, as the anthocyanin content increased up to the maximum value of 1.00%, were observed: an improvement in thickening (from 29.92 μm to approx. 37.00 μm); a decrease in wettability with water (θ goes from 14° to 31°), thus a more hydrophobic characteristic compared to pure PVA; a stiffening, as the elastic modulus increases 4-fold (from ~248 MPa to ~1060 MPa) 8° to 31.2°); a decrease in deformability, which decreases 30-fold (from ~120% to ~4%); a general decrease in tensile strength, from ~23 MPa to ~5 MPa (4.6-fold). However, looking closely at the data for the sample with 0.10% of anthocyanins, it can be seen that the presence of this small amount in the PVA improves many of the material properties: the thickness remains almost unchanged (approx. 29.00 μm); the stiffness doubles from 248 to 500 MPa; the mechanical strength increases from 23 MPa to 28 MPa; the deformability increases from 120% to 192%; the hydrophilic character remains practically unchanged, as the initial wettability value of pure PVA is approx. 15° and that of PVA + 0.10% of anthocyanins is 19°.

### 2.7. Migration Test

**Figure 5:**
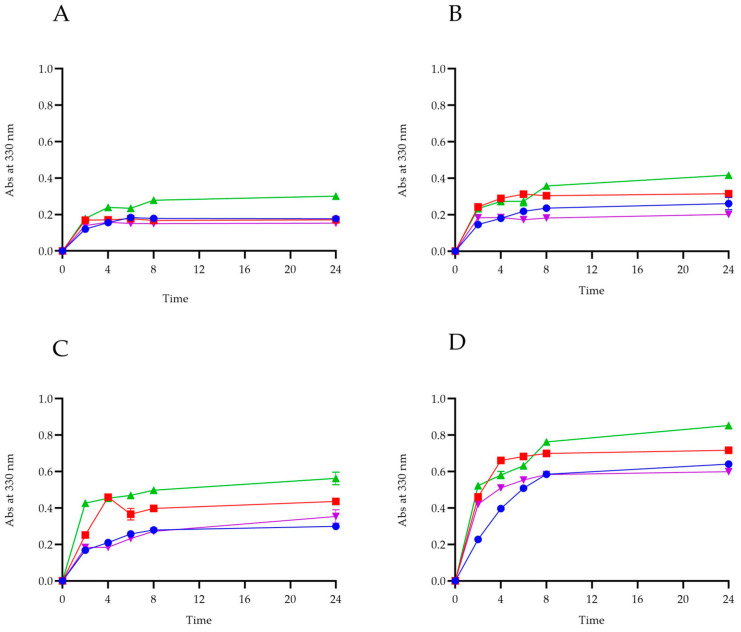
Release for short- and long-term migration of anthocyanins from PVA films in different food simulants. (**A**) PVA plus 0.10% of anthocyanins; (**B**) PVA plus 0.25% of anthocyanins; (**C**) PVA plus 0.50% of anthocyanins; (**D**) PVA plus 1.00% of anthocyanins. (⬤) H_2_O; (■) ethanol 10%; (▲) ethanol 50%; (▼) acetic acid 3%.

Using UV–visible spectroscopy, we have clearly seen how anthocyanins present within bioplastics with different % of anthocyanins are released within the different food simulants. As shown graphically (Figure 5), for each bioplastic, there is a greater release of anthocyanins in the presence of 50% ethanol. This trend is confirmed by reports in the literature, according to which the typical polyphenolic structure of anthocyanins makes these compounds very soluble in organic solvents such as ethanol and methanol [35]. Moreover, experiments carried out previously to optimize the extraction of anthocyanins from *Callistemon citrinus* powder also confirm that they solubilize more in ethanol than in totally aqueous mixtures. For each film in the Figure 5, we can clearly see how the release of anthocyanins in 50% (*v*/*v*) ethanol solution and 3% (*w*/*v*) acetic acid is more evident; this is also confirmed by experiments in the literature, from which it appears that acetic acid is used as a solvent for the extraction of anthocyanins [36]. The result obtained, showing that these compounds are easily released from the film to the different simulated food matrixes, makes it possible to preserve them from the oxidative reactions that foodstuffs may undergo, thus increasing their shelf life and contributing to produce fortified food due to the remarkable biological potential of the anthocyanins.

### 2.8. Antioxidant Activity 

**Figure 6:**
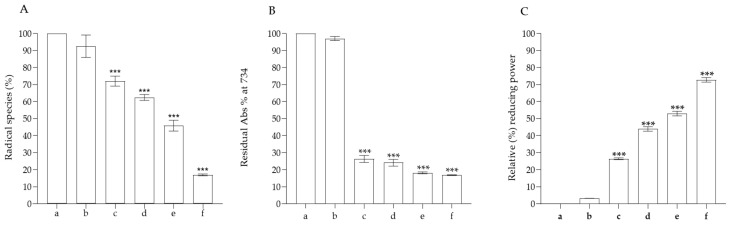
Evaluation of antioxidant activity of PVA-based bioplastics with different % of anthocyanins (0.10, 0.25, 0.50, 1%) in the most common antioxidant assays. (**A**) ABTS assay; (**B**) DPPH assay; (**C**) ferric reducing power (FRAP) assay. The letters in the different graph indicate: a, control sample; b, PVA alone; c, PVA plus 0.10% of anthocyanins; d, PVA plus 0.25% of anthocyanins; e, PVA plus 0.50% of anthocyanins; f, PVA plus 1.00% of anthocyanins. *** *p* < 0.001.

Food products packaged in different food films can be subjected to oxidation processes due to UV rays and high temperatures, causing protein denaturation and the formation of lipid peroxides, which change the organoleptic properties of the food matrix [37,38]. To understand whether our bioplastics possess antioxidant properties, we performed the most common antioxidant *abiotic* assays, such as FRAP, ABTS and DPPH. In all the assays we performed, we can clearly observe that only PVA does not exhibit any antioxidant activity, compared to bioplastics functionalized with the various % of anthocyanins. In particular, in the FRAP colorimetric assay, the reduction of ferric ion (Fe^3+^)- to ferrous ion (Fe^2+^) is used to develop an intense blue color. As shown in Figure 6, the reducing activity is proportional to the % of anthocyanins present within the bioplastic. Also, in the ABTS assay, in which a stable cationic radical is generated, we noticed that only PVA fails to have any antioxidant activity, registering an absorbance value superimposable to the blank, whereas, with the lowest % of anthocyanins, we see that there is radical scavenging activity of close to 80%. Even in the assay, in which the stable DPPH radical is generated, it can clearly be seen that the antioxidant activity is only due to the presence of anthocyanins in the functionalized bioplastics. Already, the bioplastics functionalized with 0.5% of anthocyanins were able to reduce around 50% of the radical present in solution, reaching a value of approximately 80% with the bioplastic functionalized with the highest concentration of anthocyanins. Considering the results obtained, functionalization with different % of anthocyanins was useful for providing antioxidant activity to the PVA-based bioplastics, since the latter alone do not have antioxidant activity. Indeed, the anthocyanins added to the bioplastics consist mainly of cyanidin-3,5-*O*-diglucoside and peonidin-3,5-*O*-diglucoside, which are known to possess important antioxidant activities [25,39,40]. These results suggest that the functionalization with different % of anthocyanins could confer antioxidant activity to PVA, and this can be useful for increasing the shelf-life of food that is stored and packaged, avoiding phenomena such as food rancidity.

### 2.9. Antimicrobial Activity of Anthocyanins Obtained from Callistemon citrinus

In the first part of our experiments, we tested the MIC, MFC and MBC values of the obtained anthocyanin powder against all tested strains, as shown in Table 6. These analyses were preliminary to its successively utilization for the analysis of antimicrobial activity of the functionalized bioplastic. Results of negative controls indicated the complete absence of inhibition of all the strains. Anthocyanins were active against Gram-positive bacteria, particularly *S. aureus* ATCC 6538 and *S. aureus* ATCC 43300 (MRSA), *L. monocytogenes* ATCC 13932 and *Listeria* food isolates. No effect was reported against Gram-negative strains. The most sensitive strains were *S. aureus* ATCC 43300 and *L. monocytogenes* A256, with MIC values between 0.062–0.031 and 0.250–0.500 mg mL^−1^, respectively. The observed effect was bacteriostatic rather than bactericidal. This last activity is evident only at the maximum tested concentration against *S. aureus* ATCC 43300. 

Starting from these data, we tested the potential antimicrobial activity of the functionalized bioplastics. After 24 h of incubation, the wells containing PVA functionalized with 1.00%, 0.50%, and 0.25% of anthocyanins were clear for *S. aureus* ATCC 43300, whereas the wells containing PVA at the concentration of 0.10% were cloudy, indicating bacterial growth. No activity was detected against *Listeria monocytogenes* A256. PVA at the concentration of 1.00% was bactericidal against *S. aureus* ATCC 43300. The promising result against *S. aureus* is confirmed by the other literature results: the antibacterial activity of anthocyanins is due to their effect on the bacterial membrane, increasing its permeability and favoring the escape of small soluble molecules, particularly K^+^ ions, which interrupt the proper functioning of the Na^+^/K^+^-pump and the associated metabolism. These effects of anthocyanins on the bacterial cell membrane may be ascribed to hydrogen bond formation with membrane proteins and hydrophobic interactions [41]. In addition, several experiments demonstrate a marked inhibition of protein synthesis, which, combined with the previously mentioned, drives bacterial cells to a premature programmed death [42]. *Staphylococcus aureus* and methicillin-resistant *S. aureus* (MRSA) are known to be responsible for various infections, ranging from skin, prosthesis and catheter infections to more serious systemic diseases, including endocarditis, pneumonia and osteomyelitis, as well as biofilm-associated diseases. Due to the increased resistance of *S. aureus* to antimicrobial agents, considerable effort has been focused on the development of therapeutic strategies, such as natural compounds [43]. The antibacterial activity against *L. monocytogenes* could also be due to a significant increase in membrane permeability, which, according to Sun and co-workers, leads to a leakage of DNA and various nucleic acids, a decrease in the activity of important enzymes for the bacteria, such as superoxide dismutase, adenosine triphosphate and alkaline phosphatase, and consequently the blockage of the Krebs cycle, which is necessary for the survival of the bacterium [44]. We have previously reported a bactericidal effect of *Hibiscus sabdariffa* L. anthocyanins (cyanidin-3-*O*-sambubioside and delphinidin-3-*O*-sambubioside) against a food isolate of *Listeria monocytogenes* (MBC of 2.5 mg mL^−1^) [45]. Taken on the whole and analyzing the literature, the antimicrobial effects of anthocyanins are complex and not simple to clarify. In the literature, it is possible to find a lot of studies on extracts containing anthocyanins, but very few data are available on pure compounds [46]. Obviously, the mechanism of action is different, and, in the extract, it is possible to have effects due to the phytocomplex, with synergistic and antagonistic effects that, together, collaborate to perform the described activity. On the pure compounds (e.g., pelargonidin, delphinidin, cyanidin chloride and cyanidin-3-*O*-glucoside), the effects are primary due to a destabilization of the cytoplasmic membrane and to an increase of its permeabilization, inhibition of extracellular microbial enzymes that help it to metabolize substances and nutrients, reduction of total soluble proteins (which also influence the protein synthesis process), modification of the metabolism and deprivation of essential substrates for microbial growth and reproduction, along with the anti-adherence activity, which is a prerequisite for several microorganisms’ growth [42,47,48,49].

Several biopolymers are present in the literature. Some have intrinsic antimicrobial properties, such as chitosan and polylysine. Others are not intrinsically antimicrobial in nature and require chemical incorporation of substances with antimicrobial properties, such as alginate, polylactic acid, fibrin, gelatin, starch, hyaluronic acid, cellulose, pectin and other polysaccharides [50] The produced bioplastic belongs to the former group and, upon modification with anthocyanins, acquires new antimicrobial and antioxidant activities. Moreover, as shown in the above food simulant tests, it is able to release the compounds utilized for the functionalization, making them totally available to exert their functions.

### 2.10. Food Fresh-Keeping Test

**Figure 7:**
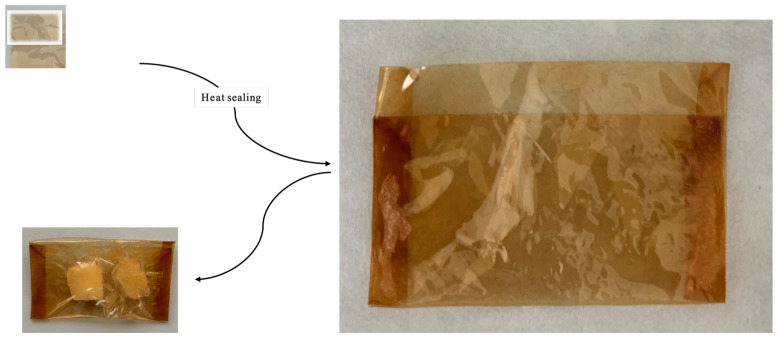
Preparation of bags for food packaging produced with PVA plus 1.0% of anthocyanins and its utilization for apple samples.

The suitability of the produced bioplastic for food packaging has been analyzed by packing fresh apple samples. Based on the data obtained in the above-described experiments, especially the mechanical and the migration tests, we decided to utilize the bioplastic functionalized with 1% of anthocyanins. We prepared bags with the bioplastic by heat sealing; a representative image of the produced bags is depicted in Figure 7. The change in the properties of the fresh apple was analyzed with a colorimeter, by the change in antioxidant activity and following browning at 420 nm after 72 h of incubation. As can be seen in Table 7, the apple samples exposed to air showed a marked browning phenomenon after 72 h of incubation, while the apple samples packed with a PVA bag functionalized with 1.00% of anthocyanins showed only limited change. In the presence of neat PVA, the samples packed show browning effects lower than the samples not packed but inferior to the effects obtained with the functionalized PVA. 

**Figure 8:**
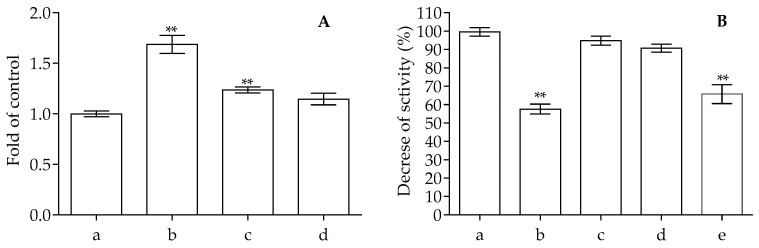
Analysis of the changes in apple samples packed or not packed after different intervals of time (0 and 72 h). (**A**) Changes in the browning of the samples monitored at 420 nm. The letters in the graph indicate: a, apple samples not packed after 0 h; b, apple samples not packed after 72 h; c, apple sample packed with PVA film alone after 72 h; d, apple samples packed with PVA plus 1.00% of anthocyanins after 72 h. (**B**) Changes in the antioxidant potential monitored by DPPH assay. The letters in the graph indicate: a, control without samples; b, apple samples not packed after 0 h; c, apple samples not packed after 72 h; d, apple sample packed with PVA film alone after 72 h; e, apple samples packed with PVA plus 1.00% of anthocyanins after 72 h. The ** indicates significant changes with respect to the control at *p* > 0.05.

The data obtained with the colorimeter were confirmed by measuring the browning reaction at 420 nm. After 72 h of incubation, there was a clear change in the absorbance of the unpacked apple samples, with an increase of about 1.68-fold respect to the control, while in the case of the packed samples, it is only 1.24- and 1.14-fold for PVA alone or functionalized with anthocyanins, respectively. The change in absorbance at 420 nm is commonly utilized to monitor non-enzymatic browning reactions, including the Maillard reaction, caramelization and the ascorbic acid browning reaction. When analyzing these products, they can be classified as small-molecule products, which contribute to food flavor and color, intermediate products, and browning compounds, which contribute to food color change [51,52]. The anthocyanins can avoid the reaction of browning by utilizing their high antioxidant activity, as shown in the above-described section, and avoiding the formation of the products of the browning reaction. The influence on the antioxidant ability is also evident in the analysis. In Figure 8, the antioxidant activity of the apple samples is depicted. The apple samples have good antioxidant activity, with the ability to scavenge about 44% of DPPH radicals, but after 72 h of incubation, this decreases to about 60% (as in the case of PVA alone), while in the samples packed with PVA plus 1.00% of anthocyanins, it is almost the same (about 40%).

## 3. Materials and Methods

### 3.1. Reagents and Standard Solutions

HPLC-grade acetonitrile, methanol, acetic acid, ethylenediaminetetraacetic acid (EDTA), sodium acetate, sodium phosphate dibasic, potassium phosphate monobasic, 2,2′-azinobis (3-ethylbenzothiazoline-6-sulfonic acid) diammonium salt (ABTS^•+^), potassium peroxydisulfate, 2,2-diphenyl-1-picrylhydrazyl (DPPH), 2,4,6-tris(2-pyridyl)-ttriazine (TPTZ), polyvinyl alcohol (PVA, Mw 89,000–98,000, 99% hydrolysis), Mueller–Hinton broth and agar were purchased from Merck (Darmstadt, Germany). Dimethylformamide (DMF) was supplied by Carlo Erba (Milano, Italy). All other chemicals and solvents used in this study were of analytical grade unless specified.

### 3.2. Optimization of the Extraction of Anthocyanins from C. citrinus and Production of PVA-Based Films 

#### 3.2.1. Sample Collection

The flowers of *Callistemon citrinus* were harvested from plant nurseries located in Messina (Italy). The flowers were dried to a moisture content of less than 2% of dry weight, ground and pulverized with a mortar, and this powder was sieved through a 250 μm mesh filter to obtain a uniform particle size and then used for anthocyanin extraction. Sieved powder was stored at −20 °C in the dark until it was used for further analysis.

#### 3.2.2. Microwave Assisted Extraction (MAE) of Anthocyanins

To extract the anthocyanins from the ground powder of *Callistemon citrinus*, we started from the protocol previously reported by Lagana et al. and modified it to produce a more environmentally friendly protocol. In the development of the experiments, we calculated the green grade of this new protocol, using the Analytical GREEnness calculator (AGREE) tools to compare this method to the starting extraction techniques [25]. Specifically, we kept the solid-to-liquid ratio of 1:10 (*w*/*v*) unchanged and replaced the previously used extraction mixture of acetic acid:methanol:water (1:70:29, *v*/*v*/*v*) with a greener mixture of acetic acid:ethanol:water (1:70:29, *v*/*v*/*v*). In addition, we optimized the extraction using microwaves, a more sustainable method than traditional methods such as maceration, as reported in the starting protocol. The mixture was subjected to microwave-associated extraction (MAE) on a CEM Discover apparatus (CEM, Matthews, NC, USA) equipped with an optical fiber (MTS-300, CEM, Matthews, NC, USA) for temperature control. Several independent variables were considered to optimize the extraction process and evaluate, through the total anthocyanin content (TAC), the best extraction conditions to obtain the highest yield. For MAE extractions, 0.100 g of ground powder was mixed in different solutions in ratio of 1:100 (*w*:*v*).

#### 3.2.3. Response Surface Methodology

Three-factor response surface methodology (RSM) was applied to an experimental design based on the Box–Behnken design (BBD) and was used to optimize the extraction conditions of anthocyanins to produce anthocyanins used to functionalize PVA-based bioplastics. As independent variables to be varied during the procedure, we selected the microwave power, the extraction time and the EtOH % concentration in the extraction mixture. For each of these independent variables, levels were chosen using a three-level, three-factor factorial BBD, which produced 15 experimental conditions, as shown in Table 1. In this experimental design, microwave power (X1) (100, 200 and 300 W), extraction time (X2) (2, 6 and 10 t), EtOH % concentration (X3) (20%, 45% and 70%) and solid/liquid ratio (S: L) fixed at 1:10 (*w*/*v*) were selected as independent factors. At the end of each experiment, we used the total anthocyanin content (TAC) as a dependent variable to evaluate the extraction conditions and the anthocyanin yield (mgL^−1^). The results obtained from the measurements of the TAC responses were fitted with a quadratic polynomial equation:

$$R=\beta 0+\beta 1X1+\beta 2X2+\beta 3X3+\beta 1,1{X1}^{2}+\beta 3,{X3}^{2}+\beta 1,2X1X2+\beta 1,3X1X3+\beta 2,3X2X3+\varepsilon$$

where R was the dependent factor (TAC) for the level of independent variables X1 (Mp), X2 (M) and X3 (EtOH%). Analysis of variance (ANOVA) was performed to evaluate significant differences and check the adjusted and predicted coefficient of determination (R2) values.

#### 3.2.4. Determination of Total Anthocyanin Content

In order to quantify the anthocyanin yield within our samples, and to evaluate the microwave extraction processes, we used the differential pH method, as reported by Giusti et al. [53,54]. This assay is based on the principle that UV–visible spectroscopy is used to measure the structural changes of anthocyanins as a result of pH changes in the solution. To perform this assay, it would be convenient to know the exact composition of the extract, to know which anthocyanin is the most representative; otherwise, it is recommended to use cyanidin-3-glucoside as the reference λvis-max. In our case, the compound most present was cyanidin-3,5-*O*-diglucoside, which has λvis-max 520. In detail, each sample extract of *Callistemon citrinus* was diluted separately in two buffers, 0.025 M hydrochloric acid-potassium chloride buffer (pH = 1) and 0.4 M sodium acetate buffer (pH = 4.5). The absorbance of each sample diluted in the respective buffers was measured at 520, which is the λvis-max of cyanidin 3,5-*O*-diglucoside, and at 700 nm, using a UV–Vis spectrophotometer (UV1601; Shimadzu, Kyoto, Japan). The total amount of anthocyanins for each sample was expressed as cyanidin-3,5-*O*-diglucoside equivalents using the following equation:

$$A=\left(A\right.\lambda vismax-A\left.700\right)pH1.0-\left(A\lambda vismax-A\left.700\right)\right.pH4.5$$

$$Anthocyanin\;pigment\left(\frac{mg}{L}\right)=\frac{A\times MW\times DF\times 1000}{\left(\varepsilon xl\right)}$$

where A is the absorbance, calculated with the first equation, MW is the molecular weight of cyanidin-3,5-*O*-diglucoside, DF is the factor of dilution used to obtain an absorbance between 0.2 and 1.2 maximum, ε is the molar absorptivity of cyanidin-3,5-*O*-diglucoside and 1.0 cm is the pathlength of our cuvette.

#### 3.2.5. Preparation of Anthocyanins and Identification of the Profile by RP-HPLC-DAD

After the optimization described above, the extract was filtered on filter paper and concentrated to a final volume of 2.0 mL via rotavapor. To obtain the anthocyanins, we performed a column separation using Supelclean™ LC-18 SPE cartridge (Supelco Ltd., Bellefonte, PA, USA). The anthocyanins were eluted with absolute ethanol and brought to dryness utilizing a watchglass for crystallization. Qualitative characterization of the anthocyanins was performed by reverse phase-high performance liquid chromatography coupled with a diode array, using an Agilent high performance liquid chromatography system (1100 series) equipped with a photodiode-array detector (DAD), following the protocol as reported by Laganà et al. [25].

#### 3.2.6. Preparation of PVA-Based Bioplastics 

PVA-based bioplastics functionalized with the anthocyanin-enriched fraction were prepared using the casting solution method [55]. In detail, 2.6 g of PVA were solubilized in 50 mL of distilled water and brought to a temperature of 90 °C for 1 h, to obtain a clear PVA solution. Subsequently, the solution was brought to a temperature of 45 °C for 30 min, and simultaneously, a solution of anthocyanins in water at various % was added for each bioplastic. Finally, 10.0 mL of each prepared solution was placed in a 9 cm diameter petri dish and left to dry for approximately 3–4 days. Thus, PVA-based bioplastics with 0.10% wt%, 0.25 wt%, 0.50 wt% and 1.00 wt% anthocyanins were obtained.

### 3.3. Characterization of PVA-Based Bioplastics Functionalized with Anthocyanins

#### 3.3.1. Fourier Transform Infrared Spectroscopy (FTIR-ATR)

Fourier transform infrared (FTIR) spectroscopy was used to evaluate whether the addition of different % of anthocyanins led to the formation of new functional groups or new interactions with PVA. The spectra were recorded with a PerkinElmer spectrometer (Paragon 500, Milan, Italy) and were performed in the region 4000–600 cm^−1^, with a resolution of 4 cm^−1^, for a total of 16 scans per sample.

#### 3.3.2. Optical and Color Properties

To determine the optical properties of bioplastics functionalized with anthocyanins, we used an UV–visible spectroscopy protocol as reported in the literature by Nascimento and coworkers [56]. For this analysis, we obtained samples of similar size and weight (1.0 × 4.0 cm) and fixed them inside a cuvette to record the transmittance (T%) for each sample at 600 nm. Subsequently, to calculate the opacity of the same samples, we compared the transmittance level to the thickness of the films (mm) (T600 nm/mm). Then, to analyze the color change of the bioplastics functionalized with anthocyanins, we used the colorimeter Eoptis CLM-194, selecting the CIE L*a*b* color space. To perform the analysis, we set an illuminant value of D65 and a visual angle of 10 °. In our case, L* varied from 0 (black) to 100 (white), a* varied from green (−) to red (+) and b* varied from blue (−) to yellow (+). As a reference, we used a white plate and calculated the color difference, using the following equation:

$$∆E=\sqrt{{\left(∆L\right)}^{2}+{\left(∆\alpha \right)}^{2}+{\left(∆\beta \right)}^{2}}$$

#### 3.3.3. Mechanical Properties and Thickness

The tensile tests of biofilms were performed according to ASTM D 638-03 standard with a 154 Lloyd LR10K Universal Dynamometer machine load cell 0.5 kN, preload 1.00 N, speed 1 mmmin^−1^ purchased from Elis–Electronic Instruments & Systems S.r.l., Rome, Italy). The tests were carried out at 25 °C and relative humidity (RH) of 30%. Mechanical parameters such as Young’s modulus (E [MPa]), load at break (Lr [N]), stress at break (σr [MPa]) and deformation at break (εr [%]) were obtained as the result of the average values obtained from eight samples (for each type).

The contact angle θw was assessed by the sessile drop method by means of the DMs-401 Contact Angle Meter instrument (KYOWA), which measures the contact angle of 2.0 μL drop of deionized water on the horizontal surface of the biofilm. The wet ability was derived from the following equation:

$$\theta_{w}=2arctg\left(\frac{2h}{d}\right)$$

To measure the thickness of biofilms in a nondestructive way, a digital thickness SAMA Tools SA8850 (SAMA Italia, Viareggio, Italy) was used. On each sample’s circular biofilm (diameter 8.0 cm), a map was drawn that identified a grid of 36 (6 × 6) points. At each point of the resulting grid, thickness measurements were made by placing the probe perpendicular to the specimen. The mean values of all measurements were then calculated.

### 3.4. Migration Test of Anthocyanins in Different Food-Simulants 

We carried out release tests to see if the anthocyanins in our biofilms were released when in contact with different foodstuffs. We worked as reported in the literature [17,57]. In detail, as stipulated by The European Standard EN. (2002), samples of our bioplastics with different % of anthocyanins were immersed in beakers containing 10.0 mL of food-mimicking solutions. In fact, to mimic the behavior and nature of different foods that may come into contact with the food bioplastics, we prepared a 100% (*v*/*v*) water solution (simulant a), a 10% (*v*/*v*) ethanol solution (simulant b), a 50% (*v*/*v*) ethanol solution (simulant c) and one with 3% (*w*/*v*) acetic acid (simulant d), in order to mimic hydrophilic, lipophilic and acidic foodstuff conditions, respectively. Samples were incubated in the different food simulants, and samplings were done at different time inter-values to assess the concentration of anthocyanins released in the solvents, with readings at 330 nm. The experiments were performed in triplicate.

### 3.5. Antioxidant Capacity

The most common antioxidant abiotic assays were performed. In particular, ferric reducing power (FRAP), 2,20-azino-bis (3-ethylbenzothiazoline-6-sulfonic acid) (ABTS) and 2,2-diphenyl-1-picrylhdrazyl (DPPH) radical scavenging assays were used to investigate the *cell-free* antioxidant capacity of the PVA-based bioplastics with different % of anthocyanins. For all the antioxidant experiments, pieces of different bioplastics with known weights (0.004 ± 0.0001 g) were cut. 

#### 3.5.1. Ferric Reducing Power (FRAP) Assay

The FRAP assay was performed following the protocol previously reported by Barreca et al. [58]. In detail, we prepared the FRAP working solution by combining a fresh solution of 2.5 mL of FeCl_3_ (20 mM), 2.5 mL of 2,4,6-Tris(2-pyridyl)-S-triazine (TPTZ) solution and 25.0 mL of acetate buffer (300 mM, pH 3.6). This solution was kept at 37 °C and monitored by absorbance reading. Then, we incubated our PVA-based samples with 1.5 mL of working solution in the cuvette with a 1.0 cm path length for 4 min and measured the absorbance at 595 nm with a spectrophotometer (BECKMAN DU 640, Brea, CA, USA). We used the working solution as a blank to calibrate the instrument. All the analyses were done in triplicate, and the results were expressed as percentages of inhibition of the radical activity. 

#### 3.5.2. Quenching of the Stable ABTS Radical 

The radical scavenging activity of our films against 2,20-azino-bis 3-ethylbenzothiazoline-6-sulfonic acid) (ABTS) was performed as reported in the literature by Barreca et al. [59]. A stable solution of the ABTS^•+^ radical was obtained by combining, the day before, a 2.45 mM solution of ammonium persulphate and a 7.0 mM of ABTS powder. The solution was left to stand overnight in the dark before use. The next day, the working solution was diluted with phosphate buffer solution to obtain an absorbance of approximately 0.80 ± 0.02 at 734 nm. Pieces of our bioplastics at various % of anthocyanins were incubated inside the cuvette with 1.0 mL of ABTS^•+^ working solution, and the absorbance was read at 0 and after 6 min at 734 nm, using a spectrophotometer. All the analyses were done in triplicate, and the results were expressed as inhibition (%) of the radical activity. 

#### 3.5.3. Inhibition of the Stable 2,2-Diphenylpyrylhydrazyl Radical (DPPH)

The DPPH antioxidant assay was performed as reported in the literature by Barreca et al. [58], with minor modifications. In detail, a fresh solution of DPPH was prepared in methanol at a final concentration of 80 μM. The DPPH radical concentration was measured in a cuvette, and dilutions in methanol were made, if necessary, until an absorbance value of about 1.0 was obtained. Pieces of bioplastics were placed in a cuvette with 1.0 mL of working solution and mixed for 10 s at room temperature. The antioxidant activity was proportional to the decrease in absorbance at 517 nm, which was recorded after 30 min of incubation. The reading was recorded using a spectrophotometer, and the analysis was repeated three times. The antioxidant activity, expressed as I (%) radical inhibition, was calculated using the following formula:

$$I\left(\%\right)=\left[\frac{Ac-As}{Ac}\right]\times 100$$

where Ac is the absorbance of the control and As is the absorbance of the sample.

### 3.6. Antimicrobial Assays

#### 3.6.1. Microbial Strains and Culture Conditions

The antimicrobial activity was investigated against a representative range of standard American Type Culture Collection (ATCC) and food isolated strains obtained from the University of Messina’s *in-house* culture collection (Messina, Italy). The test microorganisms used for antimicrobial sensitivity testing included ATCC strains of Gram-positive and Gram-negative bacteria (*L. monocytogenes* ATCC 13932, *S. aureus* ATCC 6538, methicillin-resistant *S. aureus* ATCC 43300, *P. aeruginosa* ATCC 9027, *E. coli* ATCC 10536, *S. typhimurium* ATCC 13311), the yeast *C. albicans* ATCC 10231 and four strains of *L. monocytogenes* belonging to serotypes 1/2a (1 strain), 1/2b (1 strain), 1/2c (1 strain) and 4b (1 strain). Cultures for antimicrobial activity tests were grown as previously reported [60].

#### 3.6.2. Antimicrobial Testing

The anthocyanin powder was dissolved in Mueller Hinton broth (MHB, Oxoid, UK) and Sabouraud dextrose broth (SAB, Oxoid, UK) for bacteria and yeast. In order to obtain the final concentration of 1000 µg mL^−1^, they were serially diluted up to 0.001 mgml^−1^. The minimum inhibitory concentrations (MICs) and the minimum fungicidal concentration (MFC) were determined by the broth microdilution method, according to CLSI (Clinical Laboratory Standard Institute, 2009, Clinical and Laboratory Standards Institute. CLSI. Reference Method for Broth Dilution Antifungal Susceptibility Testing of Yeasts. M27-A3. M27-A3. 4th ed. CLSI; Wayne, PA, USA: 2017). After 24 h of incubation, the MICs and MFC, expressed in µg mL^−1^, were defined as the lowest concentration of the compound that was required to completely inhibit the growth of the tested strain. 

All experiments were performed in triplicate on three independent days. The MBC (minimal bactericidal concentration) was determined by transferring each clear sample (20 μL) on an agar plate incubated at 37 °C for 24 h. The MBC was defined as the lowest sample concentration that killed 99.9% of the final inocula after 24–48 h incubation.

#### 3.6.3. Antibacterial Activity of PVA-Based Bioplastics

The PVA-based bioplastic with anthocyanins at the concentration of 1.00%, 0.50%, 0.25%, 0.10% and PVA alone were sterilized with a UV lamp for 30 min on both sides. Given the higher sensitivity of callistemon powder against *S. aureus* ATCC 43300 and *L. monocytogenes* A256, the antibacterial potential was evaluated against these strains. Small, weighed pieces of bioplastic (with a concentration of anthocyanin able to reach the tested one in the final volume) were inserted in 1.0 mL of medium (Muller Hinton broth for *Staphylococcus* and Triptic soy broth for Listeria) containing the bacteria at a concentration of 1 × 10^6^ in 24-well microplater. The microplate was incubated at 37 °C for 24 h. After incubation, 20 µL of medium were spotted from the clear wells to understand if there was any bactericidal or bacteriostatic activity.

### 3.7. Food Fresh-Keeping Test

To really see if these bioplastics can be used for food packaging and if they perform as active packaging, we carried out this assay as reported in the literature by Mirpoor and coworkers [57]. In detail, we chose apples (*Malus domestica*, variety Golden Delicious) as the food matrix. At room temperature, the apples were peeled and cut into pieces of 1.2 × 1.2 × 0.6 (L × W × H) cm with a professional stainless steel cutter with 1/2- and 1/4-inch blades, obtaining a weight of 1.5 ± 0.12 g for each apple sample. Each operation was performed under a biological fume hood to work under sterile conditions, and all the following procedures were performed in the same conditions. After cutting pieces of apple of similar size and weight, we incubated some samples without bioplastic, and others were placed in handmade packets heat-sealed with PVA alone and functionalized PVA with 1% of anthocyanins. After three days of incubation under a fume hood, the samples were used to perform colorimetric and non-colorimetric analyses to see under which conditions the freshness of the apple was most preserved. One of the most closely monitored phenomena was the browning of the sample, which was also analyzed by the digital handheld colorimeter Eoptis CLM-194 (Trento, Italia), selecting the CIE L*a*b* color space. To perform the analysis, we set an illuminant value of D65 and a visual angle of 10 °. In our case, L* varied from 0 (black) to 100 (white), a* varied from green (−) to red (+) and b* varied from blue (−) to yellow (+). As a reference, we used a white plate and calculated the color difference, using the following equation:

$$∆E=\sqrt{{\left(∆L\right)}^{2}+{\left(∆\alpha \right)}^{2}+{\left(∆\beta \right)}^{2}}$$

Subsequently, each sample was diluted 1:1 (*w*:*v*) with distilled water, subjected to homogenization and centrifuged at 12,000 rpm for 10 min, in order to take the supernatants from each sample and perform the various analyses. The supernatants taken were used to analyze the browning of each sample spectroscopically, by reading at 420 nm as reported in the literature by Xu et al., and to see the antioxidant power of each sample against the stable radical DPPH [61]. 

### 3.8. Statistical Analysis

Data are expressed as mean ± standard deviation (SD). Statistical analyses were performed by one-way analysis of variance (ANOVA). The significance of the difference from the respective controls for each experimental test condition was assayed by using Dunnett’s test for each paired experiment. A *p* < 0.05 was considered statistically significant. For mechanical data, Prism 8.0.2 statistical software (GraphPad, Inc., La Jolla, CA, USA) was used for the statistical analysis. Data are reported as mean ± SD (± standard deviation) at a significance level of *p* < 0.05. The Shapiro–Wilk test was used for normality and lognormality tests of data, and the Brown–Forsythe test for homogeneity of the variance test. Since all data used in this study satisfied these two tests, two-way analysis of variance (ANOVA) with Tukey’s post hoc test was performed to evaluate the statistical significance of the differences between the groups (significance level: 0.05).

## 4. Conclusions

This is one of the first works that focalizes on the development of a green extraction protocol to obtain anthocyanins that are utilized to functionalize class 3 bioplastics based on PVA. The results clearly show that the functionalization brings the acquisition of new functions to the newly produced bioplastics. The changes are of a morphological and mechanical nature too, because they change the transparency of the materials and make them more hydrophobic, helping to decrease one of the main problems with the utilization of PVA in food packaging applications. The acquisition of antioxidant and antimicrobial activities brings to the creation of bioplastics the ability to increase the shelf life of packed materials, especially the ones susceptible to oxidation. Moreover, the ability to release anthocyanins in the migration test can also be utilized to produce fortified food following the simple process of packaging.

## Figures and Tables

**Table 1:** Box–Behnken design with the independent factors for the recovery of the TAC from *Callistemon citrinus* powder using MAE. The values used were X1 (100, 200, 300), X2 (2, 6, 10), X3 (20, 45, 70%). TAC was expressed as mg of cyanidin-3,5-*O*-diglucoside/g.

Runs	Microwave Power (X1)	Minutes (X2)	EtOH % (X3)	TAC (mg Cyanidin-3,5-*O*-diglucoside/g)
1	200	2	70	219.275
2	300	6	20	247.105
3	100	10	45	217.755
4	200	6	45	246.835
5	300	6	70	234.170
6	300	2	45	248.660
7	100	6	20	217.755
8	100	2	45	235.515
9	200	6	45	246.835
10	200	6	45	244.235
11	200	2	20	252.305
12	200	10	20	234.170
13	100	6	70	197.625
14	200	10	70	252.610
15	300	10	45	265.280

**Table 2:** Regression Parameter Coefficients for TAC using MAE conditions. MW, microwave power (w); Minutes, extraction time (t); EtOH, % in the reaction mix (%). TAC, total anthocyanin content. * indicates significant difference (* *p* < 0.05; ** *p* < 0.01; *** *p* < 0.001).

Parameters	Regression Parameter Coefficients
	TAC (%)
MW	15.82 ***
Minutes	1.76
EtOH	−5.96 **
MW*MW	−9.80 **
Minutes*Minutes	5.83 *
EtOH*EtOH	−12.01 **
MW*Minutes	8.59 **
MW*EtOH	1.80
Minutes*EtOH	12.87 **
F-value (model)	28.45
R^2^	0.98
Adjusted R^2^	0.94
Predicted R^2^	0.70

**Table 3:** Evaluation of the optical properties of the PVA films with and without anthocyanins.

PVA + Anthocyanins	T%	Opacity (T%/mm)
PVA	89.24 ± 2.22	2.46 ± 0.90
0.1	85.67 ± 1.56	3.35 ± 0.83
0.25	82.03 ± 2.28	2.86 ± 1.10
0.5	79.40 ± 0.50	3.33 ± 1.40
1	78.91 ± 0.01	3.42 ± 0.91

**Table 4:** Colorimetric analysis of the PVA films with and without anthocyanins.

Bioplastics		L*	a*	b*	ΔE*
PVA	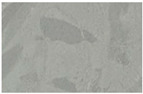	93.80 ± 0.12	−0.41 ± 0.09	2.92 ± 0.36	2.49 ± 0.22
PVA + 0.10%	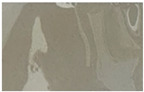	89.68 ± 1.28	−0.24 ± 0.47	13.16 ± 1.95	12.63 ± 2.31
PVA + 0.25%	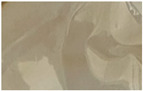	89.12 ± 1.29	0.53 ± 1.51	16.03 ± 2.28	15.52 ± 2.38
PVA + 0.50%	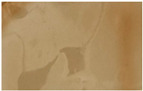	81.08 ± 3.12	4.25 ± 2.08	31.34 ± 6.70	33.02 ± 7.60
PVA + 1.0%	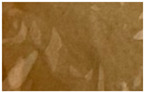	76.75 ± 2.69	6.78 ± 2.10	39.92 ± 5.69	42.90 ± 6.56

**Table 5:** Physical-mechanical properties of PVA+ anthocyanins bioplastics.

PVA + Anthocyanins [wt%]	Thickness [mm]	E [MPa]	s_b_ [MPa]	e_b_ [%]	qw [°]
0.00	29.92 ± 9.86	248.4 ± 8.5	23.76 ± 0.90	120.85 ± 6.34	14.8 ± 1.6
0.10	29.81 ± 8.04	502.7 ± 5.5	28.39 ± 0.83	192.04 ± 9.84	19.0 ± 1.1
0.25	30.57 ± 8.27	654.9 ± 7.9	17.26 ± 1.10	93.71 ± 7.12	30.2 ± 1.6
0.50	37.05 ± 9.91	800.7 ± 8.6	6.73 ± 1.40	10.67 ± 7.78	27.6 ± 1.0
1.00	36.74 ± 9.23	1060.2 ± 7.5	5.05 ± 0.91	4.09 ± 2.78	31.2 ± 2.5

**Table 6:** MICs, MBC and MFCs of anthocyanin powder against Gram-positive bacteria, Gram-negative bacteria and the yeast *C. albicans*. Values are expressed as mg mL^−1^ and represent the mean of three independent determinations.

Strain	MIC	MBC/MFC
*Staphylococcus aureus* ATCC 6538	0.500–0.250	>1.000
*Staphylococcus aureus* ATCC 43300	0.062–0.031	>1000
*Escherichia coli* ATCC 10536	>1.000	
*Pseudomonas aeruginosa* ATCC 9027	>1.000	
*Salmonella typhimurium* ATCC 13311	>1.000	
*Listeria monocitogenes* ATCC 13932	0.500	>1.000
*Listeria monocitogenes* A240 (1/2b)	1.000	>1.000
*Listeria monocitogenes* G282 (4b)	1.000	>1.000
*Listeria monocitogenes* A256 (1/2a)	0.25–0.125	>1.000
*Listeria monocitogenes* G197 (1/2c)	0.250	>1.000
*Candida albicans* ATCC 10231	>1.000	

**Table 7:** Colorimetric analysis of the apple samples packed in PVA bags with and without 1.0% of anthocyanins.

Hours		L*	a*	b*	ΔE*
Apple samples alone	0	−26.46 ± 2.96	1.76 ± 1.64	19.06 ± 0.50	32.73 ± 2.24
Apple samples alone	72	−30.62 ± 0.27	12.79 ± 0.32	37.8 ± 0.76	50.30 ± 0.60
Apple samples in PVA bags		−28.14 ± 0.14	7.40 ± 2.08	33.25 ± 0.2	44.23 ± 0.50
Apple samples in PVA +1.0% anthocyanins bags		−26.65 ± 2.23	5.41 ± 4.0	28.06 ± 2.21	39.11 ± 2.56

## Data Availability

Data are contained within the article.

## References

[1] Symeonides D., Loizia P., Zorpas A.A. (2019). Tire waste management system in Cyprus in the framework of circular economy strategy. Environ. Sci. Pollut. Res. Int..

[2] Armaghan Chizaryfard P.T.C.N. (2021). The transformation to a cirular economy: Framing an evolutionary view. J. Evol. Econ..

[3] Nordahl S.L., Scown C.D. (2024). Recommendations for life-cycle assessment of recyclable plastics in a circular economy. Chem. Sci..

[4] Visco A., Scolaro C., Facchin M., Brahimi S., Belhamdi H., Gatto V., Beghetto V. (2022). Agri-Food Wastes for Bioplastics: European Prospective on Possible Applications in Their Second Life for a Circular Economy. Polymers.

[5] Guicherd M., Ben Khaled M., Gueroult M., Nomme J., Dalibey M., Grimaud F., Alvarez P., Kamionka E., Gavalda S., Noel M. (2024). An engineered enzyme embedded into PLA to make self-biodegradable plastic. Nature.

[6] Visco A., Scolaro C., Torrisi A., Torrisi L. (2021). Diffusion of nitrogen gas through polyethylene based films. Polim. Cristallografia..

[7] Rhodes C.J. (2019). Solving the plastic problem: From cradle to grave, to reincarnation. Sci. Prog..

[8] Beena Unni A., Muringayil Joseph T. (2024). Enhancing Polymer Sustainability: Eco-Conscious Strategies. Polymers.

[9] Shaikh S., Yaqoob M., Aggarwal P. (2021). An overview of biodegradable packaging in food industry. Curr. Res. Food Sci..

[10] Beghetto V., Gatto V., Samiolo R., Scolaro C., Brahimi S., Facchin M., Visco A. (2023). Plastics today: Key challenges and EU strategies towards carbon neutrality: A review. Environ. Pollut..

[11] Elsaeed S., Zaki E., Diab A., Tarek M.A., Omar W.A.E. (2023). New polyvinyl alcohol/gellan gum-based bioplastics with guava and chickpea extracts for food packaging. Sci. Rep..

[12] Baranwal J., Barse B., Fais A., Delogu G.L., Kumar A. (2022). Biopolymer: A Sustainable Material for Food and Medical Applications. Polymers.

[13] Kalia V.C., Singh Patel S.K., Shanmugam R., Lee J.K. (2021). Polyhydroxyalkanoates: Trends and advances toward biotechnological applications. Bioresour. Technol..

[14] Prajapati R.A., Jadeja G.C. (2024). Red dragon fruit-soy protein isolate biofilm: UV-blocking, antioxidant & improved mechanical properties for sustainable food packaging. J. Food Sci. Technol..

[15] Yildirim S., Rocker B., Pettersen M.K., Nilsen-Nygaard J., Ayhan Z., Rutkaite R., Radusin T., Suminska P., Marcos B., Coma V. (2018). Active Packaging Applications for Food. Compr. Rev. Food Sci. Food Saf..

[16] Narayanasamy A., Patel S.K.S., Singh N., Rohit M.V., Lee J.K. (2024). Valorization of Algal Biomass to Produce Microbial Polyhydroxyalkanoates: Recent Updates, Challenges, and Perspectives. Polymers.

[17] Abdullah Z.W., Dong Y. (2019). Biodegradable and Water Resistant Poly(vinyl) Alcohol (PVA)/Starch (ST)/Glycerol (GL)/Halloysite Nanotube (HNT) Nanocomposite Films for Sustainable Food Packaging. Front. Mater..

[18] Wu H.F., Yue L.Z., Jiang S.L., Lu Y.Q., Wu Y.X., Wan Z.Y. (2019). Biodegradation of polyvinyl alcohol by different dominant degrading bacterial strains in a baffled anaerobic bioreactor. Water Sci. Technol..

[19] Kawai F., Hu X. (2009). Biochemistry of microbial polyvinyl alcohol degradation. Appl. Microbiol. Biotechnol..

[20] Alonso-Lopez O., Lopez-Ibanez S., Beiras R. (2021). Assessment of Toxicity and Biodegradability of Poly(vinyl alcohol)-Based Materials in Marine Water. Polymers.

[21] Barreca D., Bellocco E., Lagana G., Ginestra G., Bisignano C. (2014). Biochemical and antimicrobial activity of phloretin and its glycosilated derivatives present in apple and kumquat. Food Chem..

[22] Roselli V., Pugliese G., Leuci R., Brunetti L., Gambacorta L., Tufarelli V., Piemontese L. (2024). Green Methods to Recover Bioactive Compounds from Food Industry Waste: A Sustainable Practice from the Perspective of the Circular Economy. Molecules.

[23] Li D., Wang P., Luo Y., Zhao M., Chen F. (2017). Health benefits of anthocyanins and molecular mechanisms: Update from recent decade. Crit. Rev. Food Sci. Nutr..

[24] Ghosh D., Konishi T. (2007). Anthocyanins and anthocyanin-rich extracts: Role in diabetes and eye function. Asia Pac. J. Clin. Nutr..

[25] Lagana G., Barreca D., Smeriglio A., Germano M.P., D’Angelo V., Calderaro A., Bellocco E., Trombetta D. (2020). Evaluation of Anthocyanin Profile, Antioxidant, Cytoprotective, and Anti-Angiogenic Properties of Callistemon citrinus Flowers. Plants.

[26] Pena-Pereira F., Wojnowski W., Tobiszewski M. (2020). AGREE-Analytical GREEnness Metric Approach and Software. Anal. Chem..

[27] Li W., Wang Z., Sun Y.S., Chen L., Han L.K., Zheng Y.N. (2011). Application of response surface methodology to optimise ultrasonic-assisted extraction of four chromones in Radix Saposhnikoviae. Phytochem. Anal..

[28] Zhao L.C., Liang J., Li W., Cheng K.M., Xia X., Deng X., Yang G.L. (2011). The use of response surface methodology to optimize the ultrasound-assisted extraction of five anthraquinones from *Rheum palmatum* L. Molecules.

[29] Yang L., Jiang J.G., Li W.F., Chen J., Wang D.Y., Zhu L. (2009). Optimum extraction process of polyphenols from the bark of *Phyllanthus emblica* L. based on the response surface methodology. J. Sep. Sci..

[30] Ballard T.S., Mallikarjunan P., Zhou K., O’Keefe S.F. (2009). Optimizing the extraction of phenolic antioxidants from peanut skins using response surface methodology. J. Agric. Food Chem..

[31] Mylonaki S., Kiassos E., Makris D.P., Kefalas P. (2008). Optimisation of the extraction of olive (*Olea europaea*) leaf phenolics using water/ethanol-based solvent systems and response surface methodology. Anal. Bioanal. Chem..

[32] Kwon J.H., Belanger J.M., Pare J.R. (2003). Optimization of microwave-assisted extraction (MAP) for ginseng components by response surface methodology. J. Agric. Food Chem..

[33] Xie J.H., Xie M.Y., Shen M.Y., Nie S.P., Li C., Wang Y.X. (2010). Optimisation of microwave-assisted extraction of polysaccharides from *Cyclocarya paliurus* (Batal.) Iljinskaja using response surface methodology. J. Sci. Food Agric..

[34] Guzman-Puyol S., Benitez J.J., Heredia-Guerrero J.A. (2022). Transparency of polymeric food packaging materials. Food Res. Int..

[35] Khoo H.E., Azlan A., Tang S.T., Lim S.M. (2017). Anthocyanidins and anthocyanins: Colored pigments as food, pharmaceutical ingredients, and the potential health benefits. Food Nutr. Res..

[36] Azman E.M., Charalampopoulos D., Chatzifragkou A. (2020). Acetic acid buffer as extraction medium for free and bound phenolics from dried blackcurrant (*Ribes nigrum* L.) skins. J. Food Sci..

[37] Jatav J., Chinchkar A.V., Bhattacharya B. (2023). Chitosan film with pineapple peel extract in the extension of shelf life of Indian cottage cheese: Release kinetics and bio-accessibility studies. Food Res. Int..

[38] Bajić M., Ročnik T., Oberlintner A., Scognamiglio F., Novak U., Likozar B. (2019). Natural plant extracts as active components in chitosan-based films: A comparative study. Food Packag. Shelf Life.

[39] Hu J., Li X., Wu N., Zhu C., Jiang X., Yuan K., Li Y., Sun J., Bai W. (2023). Anthocyanins Prevent AAPH-Induced Steroidogenesis Disorder in Leydig Cells by Counteracting Oxidative Stress and StAR Abnormal Expression in a Structure-Dependent Manner. Antioxidants.

[40] Feng C., Su S., Wang L., Wu J., Tang Z., Xu Y., Shu Q., Wang L. (2016). Antioxidant capacities and anthocyanin characteristics of the black-red wild berries obtained in Northeast China. Food Chem..

[41] Lacombe A., Tadepalli S., Hwang C.A., Wu V.C. (2013). Phytochemicals in lowbush wild blueberry inactivate Escherichia coli O157:H7 by damaging its cell membrane. Foodborne Pathog. Dis..

[42] Dong Y., Yang C., Zhong W., Shu Y., Zhang Y., Yang D. (2022). Antibacterial effect and mechanism of anthocyanin from Lycium ruthenicum Murr. Front. Microbiol..

[43] Mandalari G., Minuti A., La Camera E., Barreca D., Romeo O., Nostro A. (2023). Antimicrobial Susceptibility of Staphylococcus aureus Strains and Effect of Phloretin on Biofilm Formation. Curr. Microbiol..

[44] Sun X.H., Zhou T.T., Wei C.H., Lan W.Q., Zhao Y., Pan Y.J., Wu V.C.H. (2018). Antibacterial effect and mechanism of anthocyanin rich Chinese wild blueberry extract on various foodborne pathogens. Food Control.

[45] Majdoub Y.O.E., Ginestra G., Mandalari G., Dugo P., Mondello L., Cacciola F. (2021). The Digestibility of Hibiscus sabdariffa L. Polyphenols Using an In Vitro Human Digestion Model and Evaluation of Their Antimicrobial Activity. Nutrients.

[46] de Pascual-Teresa S., Sanchez-Ballesta M.T. (2008). Anthocyanins: From plant to health. Phytochem. Rev..

[47] Nohynek L.J., Alakomi H.L., Kahkonen M.P., Heinonen M., Helander I.M., Oksman-Caldentey K.M., Puupponen-Pimia R.H. (2006). Berry phenolics: Antimicrobial properties and mechanisms of action against severe human pathogens. Nutr. Cancer.

[48] Puupponen-Pimia R., Nohynek L., Alakomi H.L., Oksman-Caldentey K.M. (2005). The action of berry phenolics against human intestinal pathogens. Biofactors.

[49] Puupponen-Pimia R., Nohynek L., Meier C., Kahkonen M., Heinonen M., Hopia A., Oksman-Caldentey K.M. (2001). Antimicrobial properties of phenolic compounds from berries. J. Appl. Microbiol..

[50] Olmos D., Gonzalez-Benito J. (2021). Polymeric Materials with Antibacterial Activity: A Review. Polymers.

[51] Adams A., De Kimpe N. (2006). Chemistry of 2-acetyl-1-pyrroline, 6-acetyl-1,2,3,4-tetrahydropyridine, 2-acetyl-2-thiazoline, and 5-acetyl-2,3-dihydro-4H-thiazine: Extraordinary Maillard flavor compounds. Chem. Rev..

[52] Kim J.S., Lee Y.S. (2008). Effect of reaction pH on enolization and racemization reactions of glucose and fructose on heating with amino acid enantiomers and formation of melanoidins as result of the Maillard reaction. Food Chem..

[53] Giusti M.M., Rodríguez-Saona L.E., Baggett J.R., Reed G.L., Durst R.W., Wrolstad R.E. (1998). Anthocyanin pigment composition of red radish cultivars as potential food colorants. J. Food Sci..

[54] Le X.T., Huynh M.T., Pham T.N., Than V.T., Toan T.Q., Bach L.G., Trung N.Q. (2019). Optimization of Total Anthocyanin Content, Stability and Antioxidant Evaluation of the Anthocyanin Extract from Vietnamese *Carissa carandas* L. Fruits. Processes.

[55] Song D., Ma L.W., Pang B., An R., Nie J.H., Guo Y.R., Li S.J. (2023). An Active Bio-Based Food Packaging Material of ZnO@Plant Polyphenols/Cellulose/Polyvinyl Alcohol: DESIGN, Characterization and Application. Int. J. Mol. Sci..

[56] do Nascimento J.V., Silva K.A., Giuliangeli V.C., Mendes A.L.D., Piai L.P., Michels R.N., Dal Bosco T.C., Ströher G.R., Shirai M.A. (2024). Starch-PVA based films with Clitoria ternatea flower extract: Characterization, phenolic compounds release and compostability. Int. J. Biol. Macromol..

[57] Mirpoor S.F., Patanè G.T., Corrado I., Giosafatto C.V.L., Ginestra G., Nostro A., Foti A., Gucciardi P.G., Mandalari G., Barreca D. (2023). Functionalization of Polyhydroxyalkanoates (PHA)-Based Bioplastic with Phloretin for Active Food Packaging: Characterization of Its Mechanical, Antioxidant, and Antimicrobial Activities. Int. J. Mol. Sci..

[58] Barreca D., Laganà G., Ficarra S., Tellone E., Leuzzi U., Galtieri A., Bellocco E. (2011). Evaluation of the antioxidant and cytoprotective properties of the exotic fruit Mill. (Annonaceae). Food Res. Int..

[59] Papalia T., Barreca D., Panuccio M.R. (2017). Assessment of Antioxidant and Cytoprotective Potential of Jatropha (*Jatropha curcas*) Grown in Southern Italy. Int. J. Mol. Sci..

[60] Bisignano C., Filocamo A., Faulks R.M., Mandalari G. (2013). In vitro antimicrobial activity of pistachio (*Pistacia vera* L.) polyphenols. Fems Microbiol. Lett..

[61] Xu Z.J., Yang Z.H., Ji J.F., Mou Y., Chen F., Xiao Z.Y., Liao X.J., Hu X.S., Ma L.J. (2023). Polyphenol mediated non-enzymatic browning and its inhibition in apple juice. Food Chem..

